# The chicken embryo as an efficient model to test the function of muscle fusion genes in amniotes

**DOI:** 10.1371/journal.pone.0177681

**Published:** 2017-05-16

**Authors:** Daniel Sieiro, Nadège Véron, Christophe Marcelle

**Affiliations:** 1 Australian Regenerative Medicine Institute (ARMI), Monash University, Clayton, Victoria, Australia; 2 Institut NeuroMyoGène (INMG), Université Claude Bernard Lyon1, Faculty of Medicine Laënnec, Lyon, France; University of Minnesota Medical Center, UNITED STATES

## Abstract

The fusion of myoblasts into multinucleated myotubes is a crucial step of muscle growth during development and of muscle repair in the adult. While multiple genes were shown to play a role in this process, a vertebrate model where novel candidates can be tested and analyzed at high throughput and relative ease has been lacking. Here, we show that the early chicken embryo is a fast and robust model in which functional testing of muscle fusion candidate genes can be performed. We have used known modulators of muscle fusion, Rac1 and Cdc42, along with the *in vivo* electroporation of integrated, inducible vectors, to show that the chicken embryo is a suitable model in which their function can be tested and quantified. In addition to nuclei content, specific characteristics of the experimental model allow a fine characterization of additional morphological features that are nearly impossible to assess in other model organisms. This study should establish the chicken embryo as a cheap, reliable and powerful model in which novel vertebrate muscle fusion candidates can be evaluated.

## Introduction

The fusion of individual myoblasts into multinucleated fibers is a crucial step in the formation, growth and repair of skeletal muscles. It is a highly dynamic process, in which cells undergo recognition, adhesion, pore formation, cytoplasmic material exchange and membrane merging [1–4]. Most of our knowledge of fusion derives from genetic analyses of the somatic musculature of the fruit fly *Drosophila melanogaster*, which have provided mechanistic insight into how myoblasts fuse with developing myofibers. In this organism, two cell populations that arise during early embryogenesis participate in muscle fusion, the founder cells (FCs) and the fusion competent myoblasts (FCMs). Each FC has a unique identity and fusion outcome, which is determined by the expression of specific transcription factors [5,6]. Individual FCs initiate muscle formation with FCMs until the final fiber size is achieved. Genetic screens performed in fly have identified about two dozen molecules that promote muscle fusion in this organism. Some of those molecules are expressed exclusively in FCs or FCMs, while others are expressed in both cell types. Recognition and adhesion between FCs and FCMs are mediated by immunoglobulin domain-containing CAMs [3]. In both cell types, their interaction triggers signaling pathways comprising molecules mainly affecting actin polymerization, resulting in the formation of invasive podosome-like structures [7,8], where the cytoplasmic material exchange between fusing cells is initiated.

Ultra- and classical microscopy analyses have shown that the cellular events that occur during myoblast fusion in *Drosophila* and vertebrates appear identical [7,9,10]. Supporting the similarity observed at a cellular level, gene knock-out studies in mouse and zebrafish have shown that the vertebrate homologs of the *Drosophila* fusion genes play the same function in those organisms [11–16]. Candidate gene approaches have led to the identification of additional modulators of fusion in vertebrates, e.g. the cell surface receptors Jamb and Jamc in fish [17] or more recently the phosphatidylserine receptors Bai1, Bai3 and stabilin-2 in mice [18–20]. An important advance was the discovery of the transmembrane molecule Tmem8c/Myomaker, which is required for muscle cell fusion in mouse and displays the remarkable property of promoting the fusion of heterologous cells to muscle cells *in vitro* [21,22].

There are, however, significant limitations to the existing vertebrate models. In mice, fusion defects are often evaluated on static tissue sections [20,22] and quantified in tissue culture [19] where conditions do not necessarily replicate those seen *in vivo*. Moreover, assaying the function of candidate genes in mouse often relies on complex, expensive and time-consuming approaches. In contrast, zebrafish are highly amenable to whole-mount and live imaging. However, muscle formation during teleost development displays numerous characteristics that reflect its evolutionary distance to mammals [23]. While biologically interesting in their own right, those peculiarities can be a serious hindrance for the translation of observations made from fish to mammals. Moreover, teleost pseudo-tetraploidy is an additional hurdle to address the genetic control of biological processes in that organism [24].

For those reasons, it would be important to develop novel, reliable and cheap experimental approaches that would allow the rapid testing of candidate genes of fusion in an amniote environment. Using the technique of *in vivo* electroporation in the chicken embryo, we have previously characterized muscle cell fusion during early embryonic development [25]. Here, we have tested whether it is a suitable and advantageous model to assay the function of candidate genes during muscle cell fusion. To test this hypothesis, we have used known regulators of fusion, Rac1 and Cdc42 [11,15,16], and a set of genome-integrated, inducible vectors that allow a time and space-restricted expression of dominant-negative forms of those transcription factors. Various approaches have been followed in recent years to show that the function of Rac1 and Cdc42 is required in muscle cell fusion during embryonic development of invertebrates and vertebrates: expression of dominant-negative forms of Drac1 and Dcdc42 in the mesoderm lineage of *Drosophila* [16]; antisense morpholinos directed against Rac1 in zebrafish [11]; conditional knock out of Rac1 and Cdc42 in the Lbx1 limb muscle lineage in mouse [15]. Here, we have targeted the expression of dominant-negative forms of Rac1 and Cdc42 to the trunk and limb muscle masses of the developing chicken embryo. We show that, similar to other species, the inhibition of Rac1 and Cdc42 function in the chicken results in a profound inhibition of fusion in the limb and in the trunk epaxial muscles. However, our approach allowed the quantification of additional cellular features of fused cells under those conditions that had not been described before (fiber length, nuclear domain, membrane irregularities, etc.). This resulted in a more comprehensive characterization of the phenotypes obtained by these fusion modulators. Altogether, we demonstrate that the chicken embryo is a good model to cheaply and reliably test the function of unknown genes involved in muscle fusion in an amniote environment, thus opening the possibility to assay large numbers of candidate genes in a relatively short time and in a cost-effective manner.

## Materials and methods

### In ovo electroporation

Fertilized “Hyline Brown” chicken eggs were obtained from Hy-Line (Bagshot, Australia) and incubated at 38°C in a humidified egg incubator. Under Australian law on animal research, experiments on fertilised eggs performed before half gestation (10 days of incubation) do not require animal ethics approval. Embryos were staged according to days of incubation. Newly formed somites were electroporated as previously described [26–28]. To target trunk epaxial muscles, interlimb somites were electroporated only in the medial border of the dermomyotome (dorso-medial lip, DML). This leads to the expression of the electroporated constructs specifically in the primary myotome. To assay limb muscle progenitor fusion, the lateral border of somites in the wing region (somites 16–21) was electroporated. This leads to the specific expression of the electroporated constructs in the limb muscle progenitors migrating into the limb bud. One day after electroporation, the embryos were examined under UV light and those that were not adequately electroporated were discarded. Electroporated embryos were analyzed after the indicated incubation times by fixing and processing for whole-mount immunostaining.

### Plasmids used for electroporation

**Fig 1:**
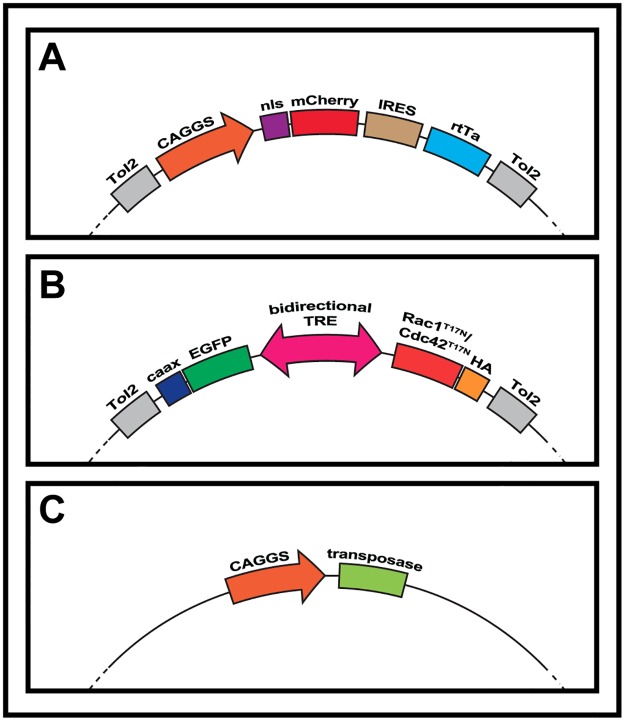
Schematic of the inducible expression system used throughout this study. Plasmids that were co-electroporated in E2.5 chicken embryos. A. The first plasmid is composed of a CAGGS ubiquitous promoter upstream of an mCherry red fluorescent protein fused to a nuclear localization signal (NLS), followed by an IRES element and an rtTa protein. B. The second construct is a bi-directional Tet-responsive element, flanked to one side by a green EGFP tagged with a CAAX box membranal signal. On the other side, a cDNA sequence (Rac1^T17N^ or Cdc42 ^T17N^) is fused to an HA tag. Both constructs are flanked by Tol2 sequences and electroporated along with a transposase plasmid (C) to ensure permanent genomic integration.

In order to create the fusion-targeting doxycycline inducible system used here, new vectors were created (see Fig 1): i) The “Tol2 CAGGS-NLSmCherry IRES rtTA advanced” plasmid (Fig 1A) was made by replacing the mEGFP from “Tol2-CAGGS-NLSCherry-IRES-mEGFP” [25]with an rtTA (reverse tetracycline-controlled transactivator protein) Advanced sequence (Clontech) used in[28] and ii) the “Tol2 pBI Rac1^T17N^ EGFPcaax” and “Tol2 pBI Cdc42^T17N^ EGFPcaax” plasmids (Fig 1B) were created by cloning Rac1^T17N^ and Cdc42^T17N^ (see below) in the bidirectional “pBI-EGFP” used in [28]. The EGFP from pBI-EGFP was replaced by a membranal EGFPcaax and this was flanked by two sequences for the Tol2 transposable elements. The resulting plasmid was called “Tol2 pBI EGFPcaax” and was used as negative control during this study. The CaaX motif from Ras present at the carboxy terminal end of GFP is a recognition sequence for a farnesyltransferase that targets GFP to the membrane. Although we observed by confocal examination that a portion of GFP is present at the membrane, the addressing to the membrane with CaaX seems rather inefficient, explaining why the GFP is also observed throughout the cytoplasm. Sequences for Rac1^T17N^ and Cdc42^T17N^ were obtained from Addgene (plasmids #15904 and #15907 respectively, [29]) and cloned in the opposite orientation to the EGFPcaax element, under the control of the CMV minimal promoter. A single human influenza hemagglutinin (HA) tag was added in-frame at the c-terminus of each coding sequence. Finally, a “CAGGS Transposase” plasmid (Fig 1C) [25] was co-electroporated to ensure genomic integration of sequences described above.

The electroporation technique results in a mosaic population of transfected cells, therefore multinucleated fibers of electroporated embryos are necessarily composed of wild type and transfected progenitors. Despite this, DAPI and RFP immunostaining showed that the nuclear mCherry marker labelled all nuclei within multi-nucleated, GFP-positive fibers. This suggests that the nuclear fluorescent marker (mCherry) is readily transferred to all nuclei within mosaic muscle fibers, indeed a crucial advantage to evaluate the number of nuclei per fiber.

### Immunohistochemistry

Embryos of the desired stages were dissected in Phosphate Buffer Saline (PBS) and fixed in 4% formaldehyde for one hour at room temperature.

For immunohistochemistry on whole mount embryos, the following antibodies were used: chicken polyclonal antibody against GFP (1:1000, Abcam); mouse monoclonal antibodies against the embryonic form of Myosin Heavy Chain MF20 (1:5, Hybridoma Bank), mouse monoclonal antibody against RFP (1:1000, Abcam) and rabbit monoclonal antibody against HA (1:200, CST). Species/isotype-specific secondary antibodies coupled with AlexaFluor-488, -555, -647 (Interchim) were used at 1:500 dilutions.

### Confocal and 3-D analysis

**Fig 2:**
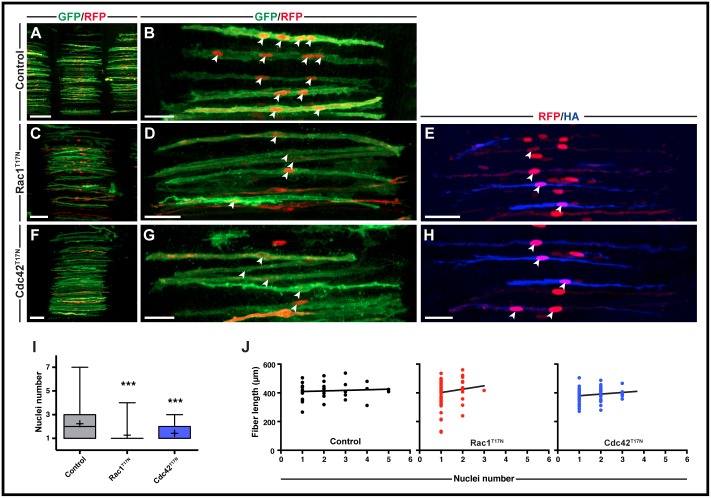
Rac1 and Cdc42 are required in early chicken trunk fusion. A-H. Somites (in dorsal view) electroporated in the DML at E2.5. Nuclei number per fiber was analyzed at E5.5. Doxycycline induction of constructs was performed at E3.5. E,H. Immunostaining with anti HA tag antibody (Rac1^T17N^ and Cdc42 ^T17N^ are both HA tagged) to ensure that doxycycline induction was efficient. **I**. Quantification of nuclei number per fiber at E5.5. Whiskers indicate minimal and maximal values; the bottom and top of the box indicate the first and third quartile; the horizontal line indicates the median; and ‘+’ indicates the mean for each set of values. **J**. Quantification of fiber length compared to nuclei number. Control: R^2^ = 0.007, "Y = 4.087*X + 404.9"; Rac1^T17N^: R^2^ = 0.016, "Y = 23.54*X + 378.3"; Cdc42^T17N^: R^2^ = 0.035, "Y = 11.34*X + 368.6". All images are whole-mount confocal stacks of fixed embryos, immunostained for EGFP (green) and RFP (red). Arrowheads (B,D,E,G,H) indicate cell nuclei within selected fibers. ***p<0.0001. Scale bars: 100μm in A,C,F; 50μm in B,D,E,G,H.

**Fig 3:**
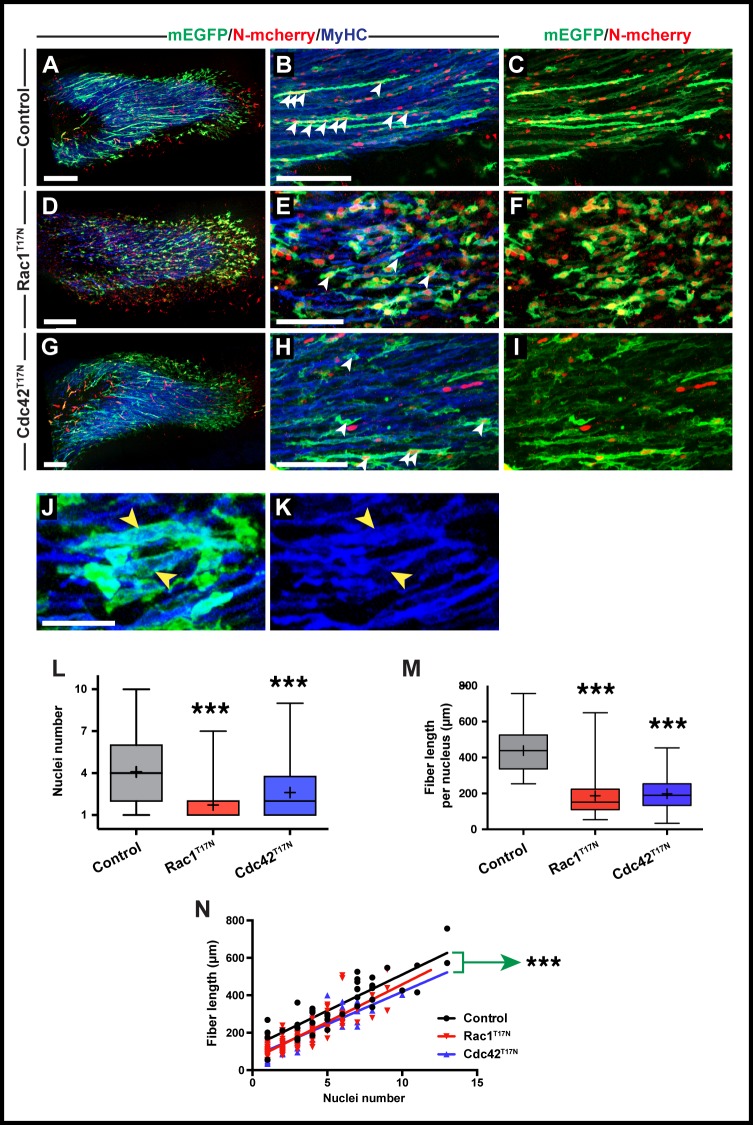
Rac1 and Cdc42 are required in early chicken forelimb fusion. A-I. Cells from the VLL at forelimb level (somites 16–21) were electroporated at E2.5 and doxycycline-expression of constructs performed from E3.5. The nuclei count per fiber was performed at E5.5. A,D,G. Confocal images of whole forelimb muscle masses. B,C,E,F,H,I. Close up of fibers within forelimb muscle masses. Arrowheads (B,E,H) indicate cell nuclei within selected fibers. J,K. Close-up of E showing that after expression of Rac1^T17N^, even GFP-positive mono-nucleated fibers (yellow arrowheads in J) express MyHC (yellow arrowheads in K). L. Quantification of nuclei number per fiber at the time points in A-I (see Fig 2 legend). M. Quantification of fiber length compared to nuclei number for all treatments. N. Compiled quantifications of fiber length compared to nuclei number for each treatment. Green arrow indicates significantly different intercept for Rac1^T17N^ and Cdc42^T17N^ compared to control. Control: R^2^ = 0.757, "Y = 38.59*X + 124.6"; Rac1^T17N^: R^2^ = 0.777, "Y = 39.75*X + 57.56"; Cdc42^T17N^: R^2^ = 0.747, "Y = 34.73*X + 71.16". All images in A-K are whole-mount confocal stacks of fixed embryos. In all images blue is MyHC, green is EGFP and red is mCherry. *p<0.05; ***p<0.0001. Scale bars: 200μm in A,D,G; 50μm in B,C,E,F,H,I,J,K.

**Fig 4:**
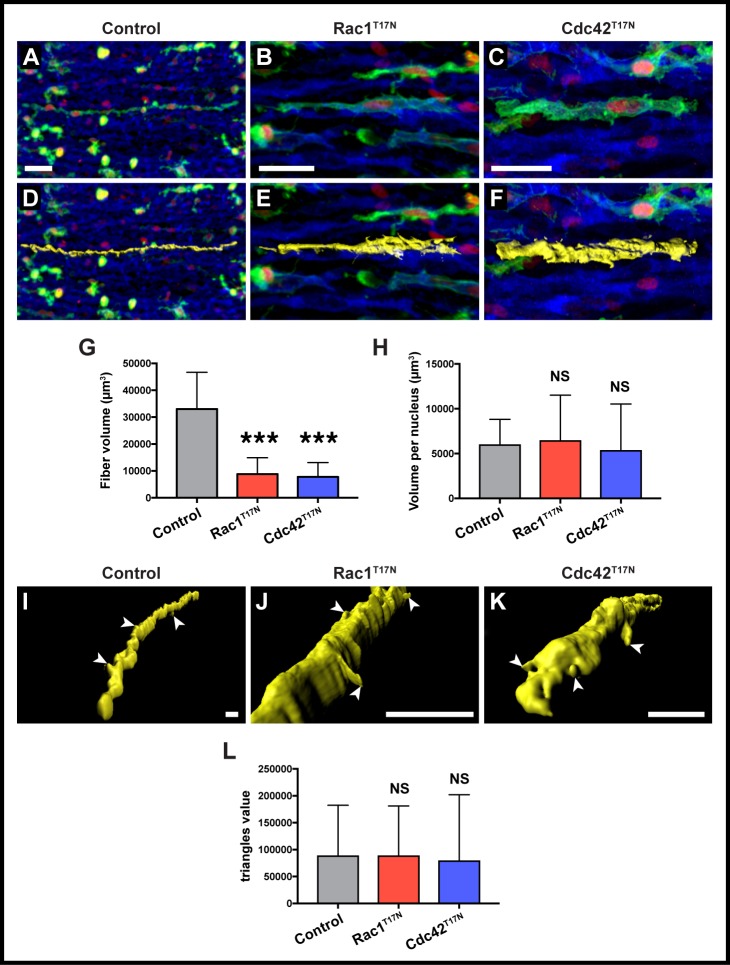
Three-dimensional rendering and analysis of limb muscle fibers. A-C. Cells from the VLL at forelimb level (somites 16–21) were electroporated at E2.5 and doxycycline-expression of constructs performed at E3.5. Confocal images of selected fibers within forelimb masses. D-F. Three-dimensional renderings of the fibers shown in A-C. Rendering are shown in yellow. G. Quantification of average total volume per fiber in control, Rac1^T17N^ and Cdc42^T17N^ positive fibers. H. Quantification of average volume per nucleus in control, Rac1^T17N^ and Cdc42^T17N^ positive fibers. I-K. Close-up of three-dimensional renderings of the fibers shown in A-C. Renderings are shown in yellow. L. Quantification of average triangles value (i.e. the number of features present in each cell surface) per fiber in control, Rac1^T17N^ and Cdc42^T17N^ positive fibers. A-F are whole-mount confocal stacks of fixed embryos. D-E and I-K show computational volume renderings of whole-mount confocal stacks of fixed embryos. In all images blue is MyHC, green is EGFP and red is mCherry. Arrowheads (I-K) indicate cellular projections on selected fibers. ***: p<0.0001, NS: p>0.05. Error bars indicate standard deviation of the mean. Scale bars: 30μm in A-C; 20μm in I-K.

Whole-mount embryos were examined using a Leica SP5 confocal microscope. Images were analyzed using Imaris and ImageJ software. The numbers obtained are presented as percentage in chart bars in Figs 2, 3 and 4. The number of nuclei in fibers at specific developmental stages was calculated as follows: mean = sumx/n, i.e. the sum of the number of nuclei in all fibers analyzed at that stage divided by the total number of fibers counted.

For 3-D analysis of volume and surface features, 20x and 40x images of GFP/MF20 immunostained, electroporated limb muscles were imaged as described above and loaded onto Imaris software. We isolated single fibers by segmentation of the region of interest based on the green (GFP) channel and then defined the sharpness of the surface, smoothing to clear the noise, and thresholding the area. We then split the whole surface to eliminate either any irrelevant particles or artifacts that remained, or other fibers that crossed it, before unifying each fragment to rebuild the surface. The software then calculated the volume area. The cell surface is determined by the software as being made up of ‘triangles’, polygonal shapes that reduce in size and increase in amount as more features are found on the surface. We therefore recorded the ‘triangles’ value as a direct readout of the state of each cell’s surface, i.e. its "roughness".

### Statistical analyses

Statistical analyses were performed using the GraphPad Prism software. Mann-Whitney non-parametric two-tail testing was applied to populations to determine P-values. In Figs 2, 3 and 4, three stars indicate P-values <0.0001, two stars: P<0.001. Furthermore, an analysis of variance (not shown) showed that the number of nuclei from different somites and different embryos within the same experimental series were not significantly different. This suggests that despite possible drawbacks of the electroporation technique (mosaicism, variability from somite to somite and from embryo to embryo, gene over-expression levels within single cells), the data obtained here are highly reproducible. For analysis of fiber length, a linear regression analysis was performed for each population to search for correlation between fiber length and nuclei number. Significant difference between slopes and intercepts of lines of best-fit between populations were also analyzed.

## Results

The *in vivo* electroporation technique we use leads to the mosaic expression of fluorescent reporters co-expressed with the genes of interest. One immediate advantage is that, using confocal microscopy, we can single out individual muscle fibers within an otherwise intricate and compact tissue. This helps the observation of muscle fibers along their entire length, and allows characterizing many of their features (nuclei number, but also fiber length, nuclear domain, membrane protrusions, etc.) in normal and mutant conditions, which in turn may allow the discovery of novel functions of candidate genes in muscles in addition to their role in fusion.

Here, we inhibited the function of the Rho GTPases Rac1 and Cdc42. We chose these molecules because they are known to be important regulators of the actin cytoskeleton and there is ample experimental evidence that they play essential roles during muscle fusion both in invertebrates and vertebrates [11,14–16]. We used dominant-negative mutants forms of Rac1 and Cdc42. The dominant-negative forms of these Rho GTPases contain a substitution of the highly-conserved threonine at position 17 to an asparagine, (Rac1^T17N^ and Cdc42^T17N^) that results in a strong and specific competition with their normal counterparts for binding to their respective GEFs [30]. Rac1^T17N^ and Cdc42^T17N^ were targeted to the trunk and limb muscle masses of the developing chicken embryo using strategies described below.

### Construction of inducible vectors to assay the function of candidate genes during fusion

We designed molecular tools that would allow testing the function of candidate genes on muscle fusion, regardless of their possible function on morphogenetic steps upstream of this process (e.g. delamination from somites, migration into the limb bud, early myogenic differentiation, etc.). We therefore opted for inducible vector strategies that could be activated at will (e.g. once progenitors have reached the limb bud mesenchyme but before they initiate fusion). We constructed two vectors, based on the Tet-on Advanced activation system (Clontech), that we have previously utilized in the chicken embryo [28,31]. The first contains the Tet-On reverse transactivator (rtTA) downstream of the CAGGS promoter, while an Internal Ribosome Entry Site (IRES) allows its expression simultaneously with a nuclear RFP variant (monomeric Cherry, mCherry, Fig 1A). The nuclear RFP is therefore constitutively expressed in these conditions. This enables testing for the efficiency of the electroporation (as the electroporated embryos are screened one day post-electroporation, see Material and methods) and allows an easy evaluation of the number of nuclei per muscle fiber at the end of the experiment. The second vector is a response plasmid in which HA-tagged dominant-negative variants of Rac1 or Cdc42 were inserted (Fig 1B). Upon addition of doxycycline, the bidirectional tetracycline-response element (TRE) drives the simultaneous expression of a membranal form of EGFP and either Rac1^T17N^ or Cdc42^T17N^. The entire functional constructs within both vectors were flanked by cis-sequences from the Tol2 transposable element [32] to allow integration into the genome of transfected cells, and thereby avoiding the gradual dilution of the plasmids with cell divisions. The integration into the genome was mediated by the co-electroporation of a third vector encoding for the Tol2 transposase enzyme (Fig 1C).

### Inhibition of early trunk muscle fusion

To test whether the dominant-negative variants of Rac1 and Cdc42 inhibit fusion in the trunk musculature, they were electroporated in the dorso-medial lip (DML) of the interlimb somites of E2.5 embryos (Fig 2). Doxycycline was added to embryos 24 hours later, shortly after initial fiber formation but prior to fusion occurring around E4.5 [25]. Embryos were collected at E5.5. Control embryos were electroporated with a response plasmid that did not contain either of the Rho GTPase.

HA-specific antibody showed that the mouse Rac1^T17N^ and Cdc42^T17N^ were highly expressed, indicating the efficiency of the doxycycline-inducible system (Fig 2E and 2H). The fusion events per fiber were determined as the number of nuclei present within each observable myofiber in confocal optical z-stacks (for more details, see Methods).

Immunostaining for GFP and RFP showed that inducible expression of Rac1^T17N^ at E5.5 almost completely blocked fusion in differentiated trunk muscle fibers since a large majority of fibers were mononucleated (mean number of 1.27 nuclei per fiber, n = 89) compared to controls (2.23 nuclei/fiber, n = 77, p<0.0001; Fig 2A–2D and 2I). Overexpression of Cdc42^T17N^ also resulted in a similarly dramatic reduction in fusion (mean number of nuclei: 1.43, n = 73, p<0.0001), with very few fibers containing more than 2 nuclei (Fig 2F, 2G and 2I). Despite Rac1^T17N^ and Cdc42^T17N^ fibers containing significantly fewer nuclei than control fibers, the length of the fibers was not different to controls (Rac1^T17N^ p = 0.42, Cdc42^T17N^ p = 0.52; Fig 2J). This is not surprising, since fibers spanned the width of the entire somite from one attachment point to the opposite regardless of their genotype.

### Inhibition of limb muscle fusion

We then tested whether Rac1^T17N^ and Cdc42^T17N^ would affect fusion in the limb muscles. Just as we can target the future trunk epaxial muscles of the chicken embryo by electroporating the DML (see above), the electroporation of the lateral portion of somites, where muscle progenitors of the forelimb are located, targets the wing muscle masses. We have previously shown that in the chicken, limb muscles undergo different fusion dynamics to those of the trunk, since fusion begins around E5 and progresses at a much faster rate than in the trunk [25].

We electroporated a mix of the three vectors (described above) in the lateral portion of somites 16–21 in E2.5 embryos (i.e. the somites from which limb muscle progenitors originate in chicken). Doxycycline was added twice to the developing embryos, once at E4 (i.e. after migration into the limb mesenchyme, but before fusion is initiated), and the second time at E5. They were then collected at E5.5. Limbs were immunostained for RFP and GFP, together with the terminal differentiation marker Myosin Heavy Chain (MyHC) to test whether inhibiting Rac1 and Cdc42 function affects myogenic differentiation.

In controls, as expected from previous studies [25], we observed that MyHC-positive electroporated fibers contained multiple nuclei (mean: 4.1, n = 128, Fig 3A–3C and 3L). In contrast, most electroporated cells present in the limbs of embryos electroporated with Rac1^T17N^ contained one or two nuclei (mean number of nuclei: 1.7, n = 224, p<0.0001, Fig 3D–3F and 3L). Despite a massive decrease of fusion of about 60%, these electroporated fibers expressed MyHC, suggesting that myogenic differentiation was not grossly affected by the loss of Rac1 function (Fig 3J and 3K).

The inhibition of Cdc42 function led to a significant, but less drastic reduction of fusion (mean number of nuclei: 2.6, n = 80, p<0.0001, i.e. 37% reduction, Fig 3G–3I and 3L). Similar to Rac1^T17N^, electroporated muscle fibers were MyHC-positive, regardless of their nuclei content (not shown). In contrast to what we observed in the trunk, the reduced number of nuclei in the limb caused by Rac1^T17N^ and Cdc42^T17N^ was accompanied by a decrease in fiber length, compared to controls (Fig 3M). This reduction in size is coherent with our previous study where we showed that in limb musculature, nuclei number and fiber length are directly correlated [25]. However, a closer look at the data showed that for a given same nuclei content, the fibers in which Rac1 or Cdc42 function was inhibited were significantly shorter than control fibers (p<0.0001, Fig 3N). This indicates that in addition to a function in the muscle cell fusion process, Rac1 and Cdc42 have a role in the growth of muscle fibers.

### Three-dimensional analysis of limb muscle fibers

The mosaic, *in vivo* expression of constructs allows an observation of single fibers that can be isolated and characterized using the power of 3-D rendering by Imaris software (S1 Movie). We tested whether 3-D visualization tools could uncover features of muscles fibers expressing dominant-negative forms of Rac1 and Cdc42 (Fig 4A–4F). We focused our attention on the ‘myonuclear domain’, defined as the amount of cytoplasm within a muscle fiber controlled by a single nucleus [33]. The nuclear domain is believed to remain constant as fiber size increases, with the addition of each nucleus accompanying an overall increase in fiber volume [34,35].

The total volume of Rac1^T17N^ and Cdc42^T17N^-expressing myofibers was significantly smaller than those of wild-type fibers (mean control: 33014.29μm^3^, n = 23; Rac1^T17N^: 8813.5μm^3^, n = 24; Cdc42^T17N^: 7775.22μm^3^, n = 23; Fig 4G). However, there was no significant difference in the myonuclear domain area per nucleus between treatments (mean control: 5934.78μm^3^; Rac1^T17N^: 6405.12μm^3^; Cdc42^T17N^: 4492.28μm^3^; Fig 4H). This suggests actin may not be playing a role in organizing nuclear domains in these early stages of fiber formation.

Additional features of individual fibers were also evaluated, such as a measurement of cell surface irregularities (named ‘triangles’, see Methods section), which uncovers differences between fibers that project many cell-membrane protrusions and extensions from those that display a ‘smoother’ cell surface (Fig 4I–4K). However, our data showed no significant difference in the amount and size of features found in the membranes of control (87843.52), Rac1^T17N^ (87729.75) and Cdc42^T17N (^78641.48) mutant fibers (Fig 4L).

## Discussion

Altogether, our data shows that the chicken embryo serves as a fast, reliable, and cheap system to test the function of candidate genes involved in muscle fusion in an amniote environment. While our data demonstrates the efficiency of the electroporation technique to achieve a robust over-expression of candidate genes, the same logic could be utilized to inhibit the function of any gene using siRNA approaches [27,28] or emerging techniques, such as the clustered regularly interspaced short palindromic repeats technology (CRISPR-Cas9 [36]). Furthermore, while the system shown here employs a ubiquitous promoter together with electroporation to physically define target populations, the use of specific promoters could further target specific cell groups and allow an even more precise analysis of fusing cells.

One definitive advantage of the experimental setup we developed in the chicken embryo over other vertebrate systems is that not only the function of muscle fusion modulators can be precisely quantified in an in vivo setting, but it also allows the evaluation of a wide range of cellular parameters and behaviors, such as fiber length, shape, cell surface irregularities, etc. This is important, because as new modulators of muscle fusion are identified, it will become essential to compare them with a variety of quantifiable criteria that may in turn give important insights into their function. Detailed description of fusion phenotypes had only been achieved before in *Drosophila*, where it was for instance observed that Kirre/Duf-null myoblasts not only did not fuse, but also projected filopodia in random directions, suggested that this protein worked as a cell attractant prior to fusion [37] In contrast, blown-fuse mutants clustered together but did not fuse, indicating that this molecule acts within the fusion machinery [9]. Similarly, our characterization of the cellular features of individual fibers after Rac1 and Cdc42 loss-of-function allowed uncovering a novel function for those molecules in fiber growth, where fibers are shorter per each nucleus when compared to controls (Fig 3N), but still retain a similar sized myonuclear domain (Fig 4H).

In conclusion, our study shows the chicken embryo has considerable potential as a reliable and powerful model on which to test potential candidate genes involved in fusion. Further studies using the methods presented here, along with novel molecules, should help elucidate this complex cellular process.

## Supporting information

S1 Movie Three-dimensional rendering of limb muscle fibers. Video showing the process of rendering a whole-mount confocal stack of fixed wild-type limb muscle fiber at E5.5 electroporated with membrane EGFP, through Imaris software. Blue is MyHC, green is EGFP and red is mCherry. Renderings are shown in yellow.
(MOV)
